# Microbial epigenetic regulation as a multilevel regulatory interface in host-microbe interactions

**DOI:** 10.1080/19490976.2026.2725428

**Published:** 2026-08-28

**Authors:** Liuel Yizengaw Adnie, Guan-Hong Wang

**Affiliations:** a State Key Laboratory of Animal Biodiversity Conservation and Integrated Pest Management, Institute of Zoology, Chinese Academy of Sciences, Beijing, People's Republic of China; b University of Chinese Academy of Sciences, Beijing, People's Republic of China; c Department of Veterinary Laboratory Technology, College of Agriculture and Natural Resources, Debre Markos University, Debre Markos, Ethiopia

**Keywords:** Epigenetic regulation, host-microbe interactions, microbial metabolites, DNA methylation, RNA regulation, tumor microenvironment

## Abstract

Animals coexist with complex microbial communities that influence their development, immunity, metabolism, and behavior. Evidence shows these effects arise not just from metabolic and immune signaling but also from epigenetic mechanisms that alter host gene expression. Microbial signals can modulate DNA methylation, histone modification, chromatin accessibility, and RNA pathways, reshaping transcription across tissues. This review synthesizes evidence from diverse animal systems to demonstrate how microbial communities influence epigenetic landscapes and contribute to immunity, development, metabolism, and neurobiology. We explore data suggesting that microbial epigenetic interactions extend into the tumor microenvironment, where intratumoral microbes may shape disease progression by remodeling epigenetic states. Comparative studies indicate that microbial regulation of host epigenetics is an evolutionarily conserved mechanism linking environmental signals to phenotype. Despite recent advances, questions remain about causality, cell-type specificity, persistence, and inheritability of these effects. We propose microbial epigenetic regulation as a key interface integrating microbial cues with host physiology and pathology, providing a framework for understanding host-microbe interactions across species.

## 1. Introduction

Microbial communities fundamentally influence animal development, metabolism, immunity, and behavior.^1^ Rather than being passive passengers, microbes play central roles in host biology through continuous molecular dialog.^2^ ^,^ ^3^ Through bidirectional signaling, microbial symbionts drive nutrient processing, support immune maturation, and maintain homeostasis, linking environmental factors to host regulation.^4^ ^,^ ^5^ Beyond these functions, microbes increasingly emerge as key modulators of host gene regulatory architecture.^6^

Epigenetic mechanisms, such as DNA methylation, histone modifications, chromatin remodeling, and RNA-based regulation, convert environmental signals into stable or semi-stable changes in gene expression without altering the DNA sequence.^7^ ^,^ ^8^ Microbial metabolites and signaling molecules act on these layers, making the epigenome a dynamic interface between microbe environment and host transcription.^6^ ^,^ ^9^ For example, short-chain fatty acids modulate histone acetylation by controlling acetyltransferases and deacetylases. Microbiota-driven immune training can also lead to long-lasting chromatin remodeling in innate immune cells.^10^ Such findings suggest microbial communities affect not only immediate physiology but also broader regulatory frameworks for long-term cellular behavior.^11^ ^,^ ^12^

Evidence shows host-microbe epigenetic interactions are conserved across animal lineages. In vertebrates, microbial metabolites help regulate immunity, metabolism, and neurophysiology through epigenetic mechanisms, enabling adaptation to changes in diet, environment, and pathogens.^13^ ^-^ ^15^ In invertebrates, microbes influence growth, reproduction, stress tolerance, and plasticity by modifying gene regulation.^16^ For instance, *Wolbachia* symbionts can alter microRNA levels, RNA methylation, and methyltransferase activity in insects, demonstrating that microbial control of host epigenetics extends beyond vertebrates.^17^ ^,^ ^18^ Such findings indicate microbial epigenetic modulation is a widespread evolutionary strategy for integrating environmental signals and maintaining adaptive responses.

Environmental pressures have shaped these interactions in different but convergent ways.^19^ In mammals, microbial regulation of epigenetics may aid adaptation to diet, immunity, and metabolic needs.^20^ In insects, short generation times and rapidly changing environments favor microbial regulation of development, nutrient use, and fitness.^21^ Comparative studies suggest microbes act as both ecological partners and regulators of phenotypic plasticity through conserved epigenetic means.^3^ ^,^ ^16^

Across animal species, microbiota-driven changes in DNA methylation, chromatin accessibility, histone modifications, and RNA regulation are increasingly observed.^22^ ^,^ ^23^ Diet-microbiota interactions show how microbial metabolism shapes host epigenetics and transcription across tissues.^6^ ^,^ ^15^ While the field is advancing quickly, much of the current evidence is associative, and key questions about mechanisms, stability, and evolutionary importance remain. The persistence and heritability of microbial-induced epigenetic states are still unclear.^24^ Thus, this review examines how microbial metabolism and signaling modulate host epigenetic regulation. We focus on immune priming, developmental plasticity, metabolic homeostasis, and neurobiology, clarifying how microbial epigenetic modulation acts as an interface at molecular, physiological, ecological, and evolutionary levels.

## 2. Epigenetic mechanisms mediating host-microbe interactions

**Figure 1.**
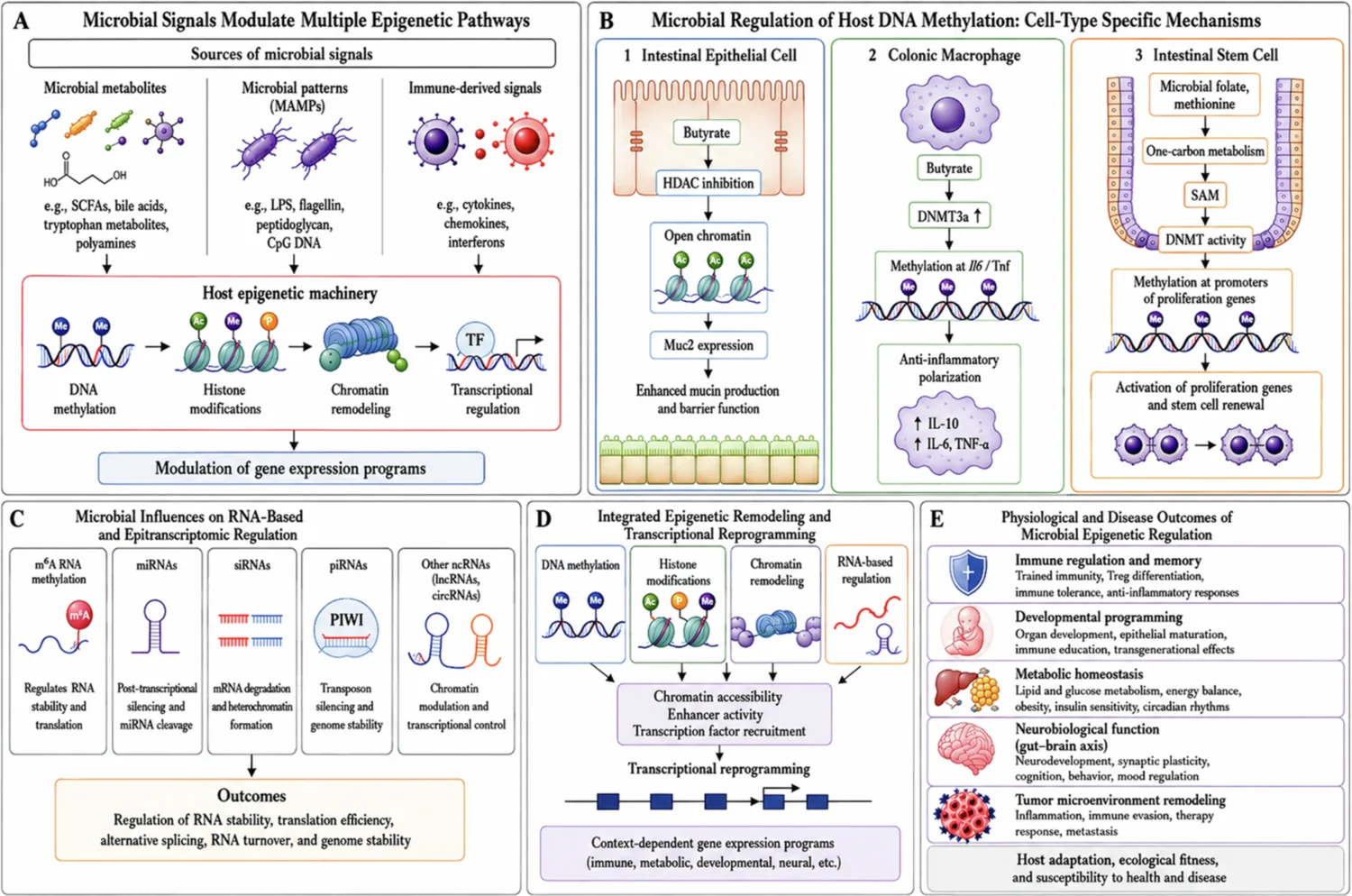
**Microbial Signals Shape Host Phenotypes Through Multilevel Epigenetic Regulation.** (**A**): How microbial metabolites, MAMPs, and immune signals modulate multiple epigenetic pathways (DNA methylation, histone modifications, chromatin remodeling, transcriptional regulation); **(B**): Cell-type-specific mechanisms of microbial regulation of host DNA methylation (intestinal epithelial cells, colonic macrophages, intestinal stem cells); (**C**): Microbial influences on RNA-based and epitranscriptomic regulation (m^6^A, miRNAs, siRNAs, piRNAs, lncRNAs/circRNAs); (**D**): Integrated epigenetic remodeling leading to transcriptional reprogramming; (**E**): Physiological and disease outcomes (immune regulation, developmental programming, metabolic homeostasis, neurological outcomes, tumor microenvironment).

Microbial symbionts influence host gene expression through convergent epigenetic pathways that couple environmental inputs to chromatin and RNA regulatory architecture.^25^ ^,^ ^26^ Rather than acting through isolated mechanisms, microbial signals interact with DNA methylation dynamics, histone modification states, chromatin accessibility, and RNA-based regulation (Figure 1A). These interactions are interconnected and context-dependent.^27^ ^,^ ^28^ Regulatory layers operate across tissues and developmental stages. Their temporal stability varies, with changes ranging from rapidly reversible transcriptional adjustments to persistent remodeling of gene regulatory networks. This multilevel organization suggests that microbial influence on host physiology occurs not only through classical signaling cascades but also through modifications of the regulatory frameworks that govern transcriptional responsiveness.^29^

### 2.1. DNa methylation and microbial regulation of host gene expression

Microbial signals regulate host gene expression through DNA methylation, a major epigenetic mechanism. In vertebrates, microbiota-associated changes in DNA methylation have been linked to intestinal homeostasis, immune regulation, and metabolic function.^13^ ^,^ ^30^ These alterations are typically locus-specific rather than global, often occurring at regulatory regions associated with inflammatory and metabolic pathways.^31^

Microbial signals affect DNA methylation in a context-dependent manner shaped by cell identity, chromatin state, and local metabolic conditions. For example, in intestinal epithelial cells, butyrate produced by *Faecalibacterium prausnitzii* acts as a class I/II histone deacetylase (HDAC) inhibitor, increasing H3K9ac and reducing DNA methylation at the *Muc2* promoter, thereby enhancing mucin production and barrier function.^9^ ^,^ ^27^ In contrast, in colonic macrophages, the same short-chain fatty acid (SCFA) signal promotes DNA methylation at the *Il6* and *Tnf* promoters via upregulation of *DNMT3*a, reinforcing anti-inflammatory polarization.^32^ In intestinal stem cells, microbiota-derived folate and methionine fuel one-carbon metabolism, supplying S-adenosylmethionine (SAM) for DNA methyltransferase (*DNMT*) activity and maintaining methylation at proliferation-associated loci.^13^ ^,^ ^30^ These cell-type-specific outcomes reflect differences in chromatin accessibility, expression of epigenetic writers/erasers, and local metabolite availability (Figure 1B). Methylation patterns vary across epithelial and immune cells, reflecting cell-specific chromatin and metabolic conditions.^13^ Early-life microbial colonization is especially influential since it coincides with high epigenetic plasticity and immune development.^33^ These developmental periods may thus mark times of greater sensitivity to microbiota-driven regulatory shifts.^34^

Microbial influences on host methylation are linked to bacterial metabolic function. In mammalian systems, members of the genera *Bacteroides*, *Bifidobacterium*, *Lactobacillus*, and *Clostridium* contribute to one-carbon metabolism by producing or modifying folate, methionine, choline, and other methyl-donor precursors required for the synthesis of SAM, the universal methyl donor used by DNA methyltransferases.^6^ ^,^ ^35^ Through these pathways, microbial communities can influence DNA methylation patterns and alter transcriptional programs associated with metabolism, immunity, and tissue homeostasis.^13^ Experimental studies further demonstrate that disruption of microbial communities through germ-free rearing or antibiotic treatment can lead to significant changes in host methylation landscapes, indicating an active role of the microbiota in epigenetic regulation^13^(Figure 1B).

Comparative analyzes reveal substantial architectural variation in DNA methylation across animal taxa. In mammals, promoter and enhancer methylation are key determinants of transcriptional regulation. Germ-free mice show altered DNA methylation at thousands of loci in colonic epithelium, particularly at genes involved in immunity and metabolism.^6^ ^,^ ^13^ In insects, DNA methylation is predominantly gene-body-enriched and is thought to stabilize transcription and regulate alternative splicing.^36^ ^,^ ^37^ Evidence for lineage and tissue-specific engagement of methylation pathways comes from multiple comparative studies. In mammals, microbiota-driven epigenetic changes are highly tissue-specific: colonic epithelial cells show DNA methylation changes at immune and barrier genes,^13^ whereas hepatic tissue exhibits epigenetic remodeling at lipid metabolism loci,^6^ and prefrontal cortex neurons display microbiota-driven transcriptional and epigenetic changes linked to social behavior.^38^ In invertebrates, *Wolbachia* infection in *A. aegypti* disrupts genome-wide patterns of cytosine methylation, impacting host methyltransferase-associated pathways ^39^; in the honey bee (*A. mellifera*), differential DNA methylation in brain tissue underlies caste-specific developmental trajectories and social behaviors.^37^ ^,^ ^40^ These comparative data collectively support the view that microbial and environmental cues engage methylation-dependent pathways in a lineage- and tissue-specific manner across evolutionarily diverse animal groups.

Mechanistically, microbial influences on DNA methylation are proposed to occur through immune signaling pathways or through modulation of one-carbon metabolism, thereby altering methyl donor availability for DNA methyltransferases.^6^ ^,^ ^13^ However, much of the current evidence remains correlative. Establishing direct causal links between defined microbial taxa, specific methylation events, and downstream phenotypic outcomes remains a major challenge. Moreover, most mechanistic insights derive from mammalian immune or epithelial models, limiting extrapolation to other taxa or whole-organism ecological contexts.

An additional unresolved question concerns the stability and heritability of microbiota-induced methylation changes. While several studies suggest that environmentally induced methylation states may persist beyond the initial exposure period and contribute to intergenerational phenotypic effects, direct evidence demonstrating stable transgenerational inheritance of microbiota-associated DNA methylation marks remains limited.^13^ ^,^ ^35^ ^,^ ^41^ This uncertainty is particularly pronounced in invertebrates, where DNA methylation is often sparse and functionally distinct from that in vertebrates.^42^ Consequently, whether microbial regulation of DNA methylation contributes to long-term inheritance of host phenotypes remains an important open question in host-microbe biology.^43^ Greater application of cell-resolved epigenomic approaches, causal microbial manipulation experiments, and broader taxonomic sampling will be necessary to determine the generality, mechanistic basis, and evolutionary significance of microbiota-associated DNA methylation dynamics.

### 2.2. RNA-based and epitranscriptomic regulation

RNA-based and epitranscriptomic mechanisms represent a dynamic regulatory layer through which microbial signals may influence host gene expression.^44^ Modifications such as N6-methyladenosine (m^6^A), together with microRNAs (miRNAs), small interfering RNAs (siRNAs), PIWI-interacting RNAs (piRNAs), and other non-coding RNAs, regulate transcript stability, translation efficiency, and decay.^8^ Emerging evidence indicates that microbial metabolites and immune-derived signals can modulate these RNA-centered pathways through several defined molecular mechanisms. First, the gut microbiota directly impacts the host epitranscriptome; microbial colonization has been shown to alter N6-methyladenosine (m^6^A) RNA methylation patterns in the cecum and liver, thereby influencing RNA stability and the translation of metabolic genes.^28^ Second, microbial tryptophan metabolites activate aryl hydrocarbon receptor (AhR) signaling pathways, which regulate mucosal immunity and influence the expression of immune-related transcripts.^45^ Third, microbial products trigger innate immune signaling, leading to epigenetic and post-transcriptional regulation, including the modulation of non-coding RNA expression and chromatin remodeling in immune cells.^11^ ^,^ ^12^ Furthermore, microbiota-derived metabolites interact with host receptors and epigenetic machinery to maintain intestinal barrier function and regulate inflammatory responses at the transcript level.^27^ Together, these mechanisms illustrate that microbial signals engage RNA and epitranscriptomic regulatory layers through direct enzymatic modulation, receptor activation, and signaling cascades (Figure 1C).

The impact of microbial cues on RNA regulation is strongly context-dependent, shaped by cell type, metabolic state, and developmental timing. For example, microbial colonization has been shown to directly impact the host epitranscriptome, altering m^6^A RNA methylation patterns in the cecum and liver, thereby influencing RNA stability and the translation of metabolic genes.^28^ Similarly, in invertebrates, *Wolbachia*-induced miRNA changes in *A. aegypti* are highly specific: the symbiont upregulates host miR-2940 to regulate transcripts of a methyltransferase, contributing to antiviral responses.^18^ Early postnatal colonization represents a period of heightened regulatory plasticity, during which microbial signals intersect with RNA-mediated and epigenetic control mechanisms. In neonatal mice, early-life microbial exposure induces persistent changes in intestinal gene expression and immune programming, establishing long-lasting immunological set points during a critical postnatal window.^46^ ^,^ ^47^ Furthermore, maternal microbial signals and metabolites can shape the neonatal epigenetic and transcriptomic landscape, linking early microbial-maternal signals to post-transcriptional and metabolic programming.^6^ ^,^ ^35^

RNA regulatory architectures vary across animal lineages. In mammals, microbiota-associated shifts are frequently linked to coordinated changes in non-coding RNA expression, enhancer activity, and RNA methylation. Microbial metabolites can influence m^6^A deposition, microRNA expression, and RNA-binding protein activity, thereby modulating immune and metabolic gene networks.^8^ ^,^ ^48^ Consequently, microbial influences on RNA processing may play a comparatively greater role in regulating transcriptional stability, developmental plasticity, and environmental responsiveness.^49^

Emerging evidence from non-model organisms further highlights the importance of non-coding RNAs in host-microbe communication, particularly through intracellular bacterial endosymbionts that manipulate host miRNA profiles. In the mosquito *A. aegypti*, *Wolbachia* infection extensively manipulates host microRNA networks to regulate epigenetic and antiviral responses. For example, *Wolbachia* upregulates host miR-2940, which targets the mRNA of the methyltransferase *Dnmt2*, linking miRNA-mediated post-transcriptional control to epigenetic reprogramming and dengue virus inhibition.^18^ ^,^ ^50^ Furthermore, *Wolbachia* induces other host miRNAs that regulate arginine methyltransferases and interfere with viral replication pathways.^51^ ^,^ ^52^ Beyond insects, studies in *Caenorhabditis elegans* demonstrate that exposure to bacteria can trigger small RNA-dependent regulatory pathways that influence immune responses and contribute to the transgenerational inheritance of environmentally induced phenotypes.^53^ These findings suggest that non-coding RNAs may function as conserved mediators of host-microbe communication across diverse animal systems.^54^

Particularly intriguing is the potential role of small RNAs in transmitting microbial information beyond the directly exposed generation. In several invertebrate systems, environmentally responsive small RNAs can persist through germline transmission and influence gene expression in offspring, providing a potential mechanism linking microbial exposure to transgenerational phenotypic effects.^24^ ^,^ ^53^ ^,^ ^55^ ^,^ ^56^ Transgenerational epigenetic inheritance in response to microbial and environmental signals is not limited to invertebrates; vertebrate systems also provide compelling evidence. In mice, paternal high-fat diet-induced microbiome dysbiosis leads to altered sperm tRNA-derived small RNAs (tsRNAs), which are transmitted to offspring and reprogram hepatic gene expression, increasing metabolic disease susceptibility.^57^ ^,^ ^58^ Similarly, gut microbial metabolism has been shown to induce transgenerational epigenetic effects; for instance, bacterial consumption of the essential nutrient choline alters host DNA methylation and metabolic phenotypes across multiple generations.^35^ Furthermore, the microbiota has been demonstrated to non-genetically modulate inherited T-cell phenotypes transgenerationally, highlighting the role of microbial signals in shaping the inherited immune landscape.^41^ Beyond direct transgenerational inheritance, early-life microbial exposure establishes persistent epigenetic and immunological set points that last throughout life. For example, microbial exposure during a critical early-life window permanently programs natural killer T (iNKT) cell function and susceptibility to inflammatory disease in offspring.^47^ These vertebrate examples, together with the invertebrate data, underscore that microbial-epigenetic transgenerational and developmental effects are broadly conserved across the animal kingdom.

Despite growing interest, much of the available evidence remains associative. Transcriptomic and epitranscriptomic profiling reveal microbiota-linked RNA alterations, yet mechanistic validation is limited, and most insights derive from mammalian cell systems.^48^ Methodologically, the field faces significant challenges, including limited cell-type resolution, which complicates data interpretation because bulk analyzes may mask important cell-specific or localized regulatory changes. This limitation hinders precise attribution of observed RNA alterations to specific microbial or host cell types, thereby reducing the robustness of causal inference. Furthermore, many studies lack phylogenetic breadth and ecological realism, as they are often confined to laboratory models rather than natural or diverse host-microbiota systems. Addressing these challenges will require broader taxonomic sampling, better integration of ecological context, and the application of advanced cell-resolved and single-cell approaches to determine the generality and functional significance of microbiota-driven RNA regulation. Together, these interconnected pathways converge to produce context-dependent chromatin remodeling and transcriptional reprogramming (Figure 1D).

## 3. Functional consequences of microbial epigenetic modulation

Epigenetic modulation provides a mechanistic bridge between microbial signals and organism-level phenotypes.^59^ By reshaping chromatin states and RNA regulatory landscapes, microbial communities influence transcriptional programs that govern immune responsiveness, developmental trajectories, metabolic homeostasis, and behavior (Figure 1E). Although converging findings suggest that microbial cues can recalibrate host regulatory set points rather than merely trigger transient responses.^6^ ^,^ ^48^ Substantial evidence for these mechanisms remains limited and often system-specific, deriving primarily from correlational studies rather than direct causal analyzes. Consequently, this perspective shifts the conceptual framework of host-microbe interactions from short-term signaling effects to integrated regulatory remodeling with potential implications for physiological stability, adaptive plasticity, and ecological fitness.^7^ ^,^ ^8^ The current understanding of these processes would benefit from further mechanistic validation and critical evaluation of existing evidence.

### 3.1. Immune regulation and epigenetic priming

One of the most extensively studied consequences of microbe-induced epigenetic regulation involves immune function. Host exposure to microbial signals can alter chromatin accessibility and transcriptional programs in innate immune cells, influencing subsequent responsiveness to infection or inflammatory stimuli. The concept of “trained immunity” illustrates how prior microbial exposure may induce epigenetic reprogramming that enhances or modulates secondary immune responses.^11^ ^,^ ^12^ ^,^ ^38^ ^,^ ^60^ Histone modifications at promoters and enhancers of inflammatory genes can persist beyond the initial stimulus, thereby shaping transcriptional potential during later challenges.

Microbiota-dependent immune regulation is not limited to acute activation. In mucosal tissues, commensal microbes contribute to immune tolerance and barrier homeostasis. Alterations in microbial composition have been associated with shifts in DNA methylation patterns and chromatin states in intestinal epithelial and immune cells, thereby influencing the inflammatory balance.^13^ ^,^ ^30^

Emerging evidence further demonstrates that microbial metabolites also influence the epigenetic programming of adaptive immune cell populations. SCFAs, secondary bile acids, and tryptophan-derived metabolites can modulate the differentiation and function of multiple T-cell subsets, including Tregs, Th17 cells, and effector CD8⁺ T cells, through alterations in histone modifications, chromatin accessibility, and transcription factor activity.^48^ ^,^ ^61^ ^,^ ^62^ Butyrate-mediated HDAC inhibition promotes *Foxp3* expression and Treg lineage stability, while microbiota-derived metabolites influence Th17 differentiation through context-dependent regulation of cytokine signaling pathways and chromatin remodeling.^63^ These epigenetic programs also contribute to the formation and maintenance of long-lasting immunological memory. During antigen exposure, epigenetic remodeling establishes permissive chromatin states at genes associated with rapid effector responses, enabling memory T cells to respond more efficiently upon secondary challenge. Microbiota-derived metabolites have been implicated in regulating the metabolic and epigenetic pathways that support memory T-cell differentiation and persistence, including pathways linked to mitochondrial metabolism, histone acetylation, and enhancer accessibility.^64^ ^,^ ^65^ Collectively, these findings illustrate how microbial epigenetic modulation shapes adaptive immunity across multiple levels of immune organization. Although the precise mechanisms remain incompletely understood, these observations suggest that microbial communities may influence not only immediate immune responses but also the long-term adaptive immune landscape.^66^

While these findings support a role for metabolite-driven epigenetic remodeling in both innate and adaptive immunity, important questions remain. It is unclear whether microbial metabolites act directly on T cells or indirectly through antigen-presenting cells and other stromal populations. Furthermore, the extent to which microbiota-induced epigenetic changes persist over time and contribute to durable immune memory remains unresolved. Future studies combining cell-specific epigenetic manipulation, single-cell multi-omics, and longitudinal immune profiling will be necessary to distinguish stable lineage commitment from transient functional modulation. Collectively, these findings illustrate how microbial epigenetic modulation influences immune responsiveness across multiple levels of immune organization, linking microbial ecology to both immediate host defense and long-term immune memory.^67^

### 3.2. Developmental programming and plasticity

In both vertebrates and invertebrates, epigenetic regulation contributes to developmental plasticity by integrating environmental information into gene expression programs that govern growth, differentiation, and physiological function.^37^ ^,^ ^40^ Among environmental factors, microbial symbionts represent an important source of developmental signals. Through interactions with nutritional, endocrine, and immune pathways, microbiota can influence host developmental trajectories and tissue maturation.^6^ ^,^ ^48^ Microbial metabolites and signaling molecules have been implicated in regulating developmental gene networks through epigenetic mechanisms, including DNA methylation, histone modification, chromatin remodeling, and RNA-based regulation.^68^ In insects, DNA methylation and histone modifications contribute to phenotypic differentiation, developmental stability, and alternative splicing.^36^ ^,^ ^37^ In the honey bee (*A. mellifera*), differential DNA methylation in brain tissue underlies caste-specific developmental trajectories between queens and workers, demonstrating how epigenetic mechanisms regulate social phenotypes.^37^ Beyond honey bees, comparative studies reveal that DNA methylation patterns vary across insect taxa and are associated with phenotypic plasticity and environmental responsiveness.^36^ ^,^ ^40^ In *A. aegypti*, *Wolbachia* infection disrupts genome-wide cytosine methylation patterns and modulates host methyltransferase-associated pathways, linking microbial symbionts to epigenetic reprogramming and antiviral responses.^18^ ^,^ ^39^ Furthermore, *Wolbachia* has been shown to alter the m^6^A methylation landscape and elevate host methyltransferase expression in mosquito cells, demonstrating microbial control of the host epitranscriptome.^17^ These findings illustrate that microbial inputs influence insect developmental and physiological outcomes through specific epigenetic mechanisms, including DNA methylation, histone modifications, and RNA-based regulation.^54^ In vertebrates, microbiota-dependent epigenetic programming has been linked to immune maturation, intestinal development, and metabolic regulation during critical developmental windows.^46^

#### 3.2.1. Prenatal and early-life microbial epigenetic programming

Although the fetal environment was historically considered sterile, accumulating evidence indicates that maternal microbiota-derived metabolites and microbial-associated molecular signals can reach the developing fetus during pregnancy and influence epigenetic programming during critical developmental windows. Rather than supporting persistent fetal colonization, current evidence suggests that maternal microbial products, including SCFAs, AhR ligands, and other microbial metabolites, contribute to fetal immune and metabolic development through epigenetic mechanisms.^6^ ^,^ ^45^ ^,^ ^46^ These metabolites regulate DNA methylation, histone modifications, chromatin accessibility, and transcriptional programs that establish long-term developmental trajectories.

Among these microbial metabolites, SCFAs, particularly butyrate and acetate, act as endogenous inhibitors of HDACs, promoting histone acetylation and enhancing *Foxp3* expression to facilitate regulatory T-cell differentiation and immune tolerance.^48^ ^,^ ^61^ Complementing these findings, early-life and maternal microbial exposure have been shown to prime innate immune development in offspring through microbiota-derived signals that establish long-lasting immunological set points.^46^ ^,^ ^47^ Furthermore, the gut microbiota modulates the availability of one-carbon metabolites, including choline and other methyl-donor precursors, which regulate SAM-dependent DNA methylation and contribute to long-term metabolic and transgenerational programming.^6^ ^,^ ^35^ Together, these studies indicate that maternal microbial function provides an early environmental cue that shapes fetal and neonatal epigenetic landscapes and developmental plasticity, thereby influencing immune competence, metabolic homeostasis, and disease susceptibility throughout life (Table 1).

**Table 1.** Examples of mechanisms of prenatal microbial-epigenetic regulation during fetal development.

Maternal/Early-life microbial signal	Route of transfer/Timing	Fetal/Neonatal target tissue	Epigenetic mechanism	Functional outcome	References
SCFAs (butyrate, propionate)	Placental circulation/Early postnatal	Fetal thymus, gut, colon	HDAC inhibition → H3K9ac at *Foxp3* enhancer	Priming of regulatory T-cell (Treg) development and immune tolerance	[48,61]
Choline/One-carbon metabolites	Placental one-carbon pool/Prenatal	Fetal liver, metabolic tissues	SAM-dependent DNA methylation at metabolic loci	Growth, metabolic programming, and transgenerational effects	[35]
Tryptophan metabolites (Indoles)	Placental circulation/Postnatal	Fetal gut, immune cells	AhR signaling → chromatin remodeling at immune loci	Immune tolerance and mucosal homeostasis	[45]
Early-life microbial exposure Microbial-Associated Molecular Patterns (MAMPs)	Maternal-fetal interface/Critical postnatal window	Hematopoietic cells, iNKT precursors	Epigenetic imprinting of inflammatory loci	Persistent programming of innate immune (iNKT) cell function	[46,47]
Microbial metabolites (Circadian)	Systemic circulation/Postnatal	Liver, metabolic tissues	HDAC3 recruitment → rhythmic histone acetylation	Diurnal programming of hepatic lipid and glucose metabolism	[69]
Microbial signals	Postnatal colonization	Intestine, Liver	Modulation of m^6^A epitranscriptome	Post-transcriptional immune and metabolic regulation	[28]

Beyond structural development, microbial epigenetic interactions have been proposed to influence the maturation of physiological systems, including the immune, metabolic, and nervous systems.^70^ Early-life microbial colonization coincides with periods of heightened epigenetic plasticity, during which microbial signals may establish long-lasting transcriptional programs that influence physiological function throughout life.^47^ However, the extent to which these changes represent stable developmental reprogramming rather than reversible adaptive plasticity remains unresolved.^71^ Longitudinal studies across developmental stages are therefore needed to distinguish transient regulatory adjustments from persistent epigenetic imprinting.

### 3.3. Metabolic homeostasis and nutrient sensing

Metabolic regulation represents another major domain in which microbial epigenetic modulation has been documented. Microbial communities influence host nutrient availability and generate metabolites that directly affect chromatin-modifying enzymes and transcriptional regulators responsive to cellular metabolic states.^20^ Consequently, changes in microbial composition can alter host epigenetic landscapes and metabolic gene expression programs.^6^ ^,^ ^22^

Among the best-characterized microbial metabolites are SCFAs, including acetate, propionate, and butyrate. These molecules function not only as energy substrates but also as epigenetic regulators that modulate the activities of histone acetyltransferases and histone deacetylases. Through these mechanisms, SCFAs influence chromatin accessibility and transcriptional activity at genes involved in lipid metabolism, glucose homeostasis, and inflammatory regulation.^6^ Diet-induced shifts in microbiota composition can therefore reshape histone acetylation patterns and enhancer activity within metabolic tissues, leading to altered metabolic phenotypes.^72^

#### 3.3.1. Microbial epigenetic regulation of hepatic physiology

The liver is a central metabolic organ that undergoes extensive structural and functional maturation during early life and is increasingly recognized as a major target of microbiota-derived epigenetic regulation. Gut microbial metabolites, including SCFAs, secondary bile acids, and other microbial products, reach the liver through the portal circulation, where they regulate hepatocyte function by modulating DNA methylation, histone modifications, chromatin accessibility, and RNA-mediated regulatory pathways. SCFAs, particularly butyrate and propionate, function as HDAC inhibitors, promoting histone acetylation and transcriptional activation of genes involved in lipid oxidation, gluconeogenesis, and glucose homeostasis.^73^ ^,^ ^74^ In addition, microbial metabolism of primary bile acids generates secondary bile acids that activate the nuclear receptor farnesoid X receptor (FXR) and the G protein-coupled bile acid receptor (TGR5), thereby regulating chromatin remodeling and transcriptional programs controlling bile acid synthesis, lipid metabolism, and hepatic inflammation.^75^ ^,^ ^76^ The intestinal microbiota also coordinates circadian hepatic metabolism through histone deacetylase 3 (HDAC3), which rhythmically remodels chromatin accessibility and regulates metabolic gene expression in response to microbial and nutritional cues.^69^ Beyond chromatin regulation, microbial colonization shapes the hepatic epitranscriptome by altering m^6^A RNA methylation, thereby influencing RNA stability and translation of metabolic genes involved in lipid and carbohydrate metabolism.^28^ Furthermore, microbial metabolites maintain intestinal barrier integrity and limit hepatic inflammation by regulating epithelial and immune-cell chromatin states, thereby reducing translocation of inflammatory microbial products through the gut-liver axis.^27^ Collectively, these findings identify the liver as a central hub of microbial-epigenetic communication, where early-life microbial colonization establishes long-lasting epigenetic programs that influence metabolic homeostasis and susceptibility to metabolic disorders, including non-alcoholic fatty liver disease, obesity, and insulin resistance.

In models of obesity and metabolic dysfunction, microbiota-associated changes in chromatin accessibility have been observed in regulatory regions that control energy balance and inflammatory pathways.^77^ These findings suggest that microbial signals may be associated with metabolic phenotypes not only through direct biochemical interactions but also by influencing gene regulatory architecture. Similarly, microbiota-dependent modulation of histone deacetylase activity contributes to circadian regulation of metabolism, linking microbial rhythms to host transcriptional oscillations and metabolic homeostasis.^15^ ^,^ ^69^ Despite growing evidence linking microbial composition to metabolic epigenomic states, disentangling direct microbial effects from secondary metabolic feedback remains challenging. Alterations in diet, energy balance, inflammation, and host physiology can independently influence epigenetic landscapes, complicating causal inference. Controlled experimental systems that manipulate microbial communities independently of dietary variables will therefore be essential for identifying direct mechanistic pathways. Furthermore, whether microbiota-associated metabolic epigenetic changes persist beyond immediate exposure or contribute to longer-term physiological programming remains largely unresolved.^77^

### 3.4. Neurobiological outcomes and the gut-brain epigenetic axis

Emerging evidence suggests that microbial influences on epigenetic regulation extend beyond peripheral tissues to affect neurodevelopment, cognition, and behavior.^78^ The gut-brain axis provides a conceptual framework for understanding how microbial metabolites, immune mediators, endocrine factors, and neural signaling pathways interact to influence central nervous system (CNS) function.^38^ ^,^ ^60^

A growing body of evidence indicates that microbiota-derived metabolites serve as molecular intermediates linking intestinal microbial communities to epigenetic regulation in the brain.^79^ Among these, SCFAs are particularly important because they can enter the systemic circulation and cross the blood-brain barrier via monocarboxylate transporter systems ^80^ ^,^ ^81^ and by inhibiting histone deacetylases, thereby increasing histone acetylation and enhancing transcription of genes involved in neuronal plasticity, synaptic function, and neuroprotection.^82^

One notable target is brain-derived neurotrophic factor (*BDNF*), a critical regulator of neuronal development, learning, and memory. Increased histone acetylation at *BDNF* regulatory regions has been associated with microbiota-dependent transcriptional activation and improved neuronal function.^38^ ^,^ ^83^ Through these mechanisms, microbial metabolites may directly remodel transcriptional programs in neurons and glial cells.^84^

Additional microbial signaling molecules also contribute to neuroepigenetic regulation. Microbiota-dependent metabolism of dietary tryptophan generates indole derivatives that activate AhR signaling pathways in both immune and neural cells.^45^ These pathways influence chromatin remodeling, transcription factor recruitment, and neuroimmune communication. Similarly, microbiota-modified bile acids have been implicated in regulating gene expression networks involved in neuroinflammation and neuronal signaling.^85^

Microbial effects on the CNS may also occur indirectly through immune-mediated mechanisms. The microbiota plays a critical role in microglial maturation and function, and microbial depletion alters microglial transcriptional profiles and impairs immune responsiveness in the brain.^83^ Changes in chromatin accessibility and histone modification states within microglia suggest that microbiota-dependent epigenetic remodeling contributes to long-term regulation of neuroimmune homeostasis.^86^

RNA-based mechanisms may provide an additional layer of regulation. Specific RNA-centered mechanisms linking microbial signals to neuronal gene regulation, behavioral plasticity, and cognitive function, including: (i) microRNA modulation: microbiota depletion in mice alters the expression of specific microRNAs (including miR-137 and miR-124) in the prefrontal cortex and hippocampus, which target transcripts involved in synaptic plasticity and are associated with anxiety-like and social behavior phenotypes ^87^; (ii) epitranscriptomic and transcriptomic regulation: dynamic RNA modifications and splicing events are critical for brain development, and emerging evidence demonstrates that the gut microbiome directly influences these neuroepigenetic pathways and transcriptomes ^38^ ^,^ ^88^; (iii) alternative splicing regulation: germ-free mice show altered RNA splicing in the amygdala, with microbiota-dependent changes affecting transcripts involved in social behavior^88^; and (iv) exosomal RNA transfer: microbiota-derived extracellular vesicles and other systemic factors can cross the blood-brain barrier and modulate neuronal gene expression, influencing neuroinflammation and cognitive function.^87^ These RNA-centered mechanisms converge with chromatin-level regulation to shape neurobiological outcomes.

Compared with evidence in the immune and metabolic domains, evidence for direct epigenetic mediation of behavioral outcomes remains relatively limited.^6^ ^,^ ^13^ ^,^ ^28^ Nevertheless, accumulating studies demonstrate that the gut microbiota is associated with changes in neural gene expression, chromatin accessibility, and epigenetic regulatory pathways, suggesting that it participates in shaping neurobiological phenotypes through multiple convergent mechanisms.^89^ The epigenetic mediation of social behavior is particularly well-studied in insects, where microbial signals intersect with conserved epigenetic mechanisms to shape social phenotypes. In the honey bee (*A. mellifera*), differential DNA methylation in brain tissue regulated by the Dnmt3 underlies caste-specific developmental trajectories and social behaviors; royal jelly components inhibit HDAC activity, increasing histone acetylation and altering DNA methylation to promote queen differentiation.^37^ ^,^ ^90^ In *Drosophila melanogaster*, gut microbiota influence social behaviors, including mating preference, through epigenetic and transcriptomic mechanisms; commensal bacteria alter host gene expression profiles that drive assortative mating and pheromone perception.^60^ In ants (*Camponotus floridanus*), epigenetic reprogramming, including DNA methylation and histone modifications, regulates caste-specific behavior and division of labor between major and minor workers, linking genomic regulation to complex social structures.^40^ ^,^ ^91^ These examples illustrate that microbial-epigenetic interactions are integral to the regulation of social behavior across insect taxa. Future integration of metabolomics, single-cell epigenomics, spatial transcriptomics, and neurophysiological approaches will be essential for establishing causal links between microbial communities, epigenetic remodeling, and behavioral outcomes.

Across these physiological domains, microbial signals influence host regulatory architecture at multiple levels, generating context-dependent effects on development, metabolism, immunity, and behavior.^6^ ^,^ ^15^ Some microbiota-associated epigenetic changes appear to be transient and reversible, whereas others may persist beyond the immediate exposure and contribute to long-term physiological recalibration.^46^ ^,^ ^47^ ^,^ ^92^ ^,^ ^93^ Understanding these temporal dynamics remains central to determining whether microbial epigenetic modulation primarily supports adaptive plasticity, durable developmental programming, or both. Framing these interactions within broader ecological and evolutionary contexts highlights how microbial cues are integrated into host regulatory systems, influencing fitness, adaptation, and health across diverse environments.^31^

### 3.5. Intratumoral microbial heterogeneity and epigenetic remodeling in disease microenvironments

Recent studies have expanded the concept of host-microbe interactions beyond traditional mucosal environments to include microbial communities residing within tumor tissues. A growing body of evidence indicates that tumors harbor distinct and heterogeneous microbial populations that contribute to the complexity of the tumor microenvironment. Gao et al^94^ highlighted that intratumoral microbial heterogeneity represents an important but often overlooked component of tumor biology, influencing cellular diversity, disease progression, and therapeutic responses through interactions with host regulatory pathways.

Microbial communities within tumors may generate localized metabolic and signaling environments that influence host epigenetic machinery. Different microbial taxa produce distinct metabolites, including short-chain fatty acids, polyamines, and secondary bile acids, which can affect DNA methylation, histone modifications, chromatin accessibility, and RNA-mediated regulation. Through these mechanisms, intratumoral microbes may alter transcriptional programs associated with cell proliferation, apoptosis, angiogenesis, immune evasion, and metastatic progression.^94^ ^,^ ^95^

The influence of intratumoral microbes extends beyond malignant cells to immune and stromal compartments of the TME. Microbial metabolites and microbe-associated molecular patterns can promote epigenetic remodeling in macrophages, dendritic cells, T lymphocytes, and fibroblasts, thereby shaping inflammatory responses and immune surveillance. Such alterations may contribute to the establishment of immunosuppressive microenvironments that facilitate tumor progression and resistance to therapy.^94^ ^,^ ^96^

Evidence for tumor-associated microbial heterogeneity has been strengthened by large-scale profiling studies demonstrating that different cancer types harbor distinct intracellular bacterial communities.^97^ These findings suggest that microbial composition may represent an additional layer of biological variability within tumors and may contribute to inter-patient differences in disease progression and treatment outcomes. Furthermore, microbial influences on chromatin accessibility and transcriptional regulation have been proposed as mechanisms by which the intratumoral microbiota affect responses to chemotherapy, radiotherapy, and immunotherapy.^98^ ^,^ ^99^

Collectively, the tumor microenvironment provides a compelling example of how microbial communities can regulate host gene expression through epigenetic pathways.^100^ Incorporating intratumoral microbial ecology into the broader framework of microbial epigenetic regulation expands our understanding of the microbe-host interface beyond normal physiology and highlights its relevance in complex disease states.^100^ Future integration of metagenomics, metabolomics, spatial transcriptomics, and single-cell epigenomics will be essential for elucidating how microbial heterogeneity contributes to disease development through epigenetic remodeling of host cellular networks.

## 4. Emerging challenges and future perspectives in microbial epigenetic regulation

### 4.1. From correlation to mechanism

A major challenge in microbial epigenetics is distinguishing causal regulatory interactions from associative relationships. Numerous studies report concurrent alterations in microbial composition and host epigenetic states, yet direct mechanistic evidence remains limited. For example, microbiota-derived SCFAs can influence histone acetylation and gene expression in host tissues, while early-life microbial colonization has been linked to persistent immune reprogramming.^6^ A notable example is the study by,^47^ which demonstrated that germ-free mice exhibit persistent alterations in invariant natural killer T-cell function that can only be corrected during a critical early-life colonization window. This finding illustrates how microbial exposure can induce durable immunological and epigenetic consequences extending beyond the immediate period of microbial contact. However, identifying the specific microbial taxa, metabolites, and host regulatory pathways responsible for these effects remains a central objective of the field. Future integration of gnotobiotic models, metabolomics, and functional epigenomics will be critical for establishing causality.

### 4.2. Temporal stability and biological significance

An important unresolved question concerns the persistence of microbiota-associated epigenetic modifications.^13^ Some regulatory changes appear transient and closely coupled to microbial presence, whereas others may persist long after the initiating exposure. Studies in mammals suggest that early-life microbial colonization can establish durable immune and metabolic phenotypes through epigenetic remodeling.^46^ ^,^ ^47^ Nevertheless, the extent to which these changes represent stable epigenetic memory, developmental plasticity, or reversible adaptation remains unclear. Resolving these temporal dynamics is essential for understanding the long-term biological significance of host-microbe epigenetic interactions.^101^

### 4.3. Cell-specific and tissue-specific regulatory networks

Microbial signals influence diverse tissues through distinct epigenetic mechanisms. In the immune system, SCFAs promote regulatory T-cell differentiation by epigenetically regulating the *Foxp3* locus.^48^ ^,^ ^61^ In metabolic tissues, microbiota-dependent chromatin remodeling contributes to the regulation of lipid and glucose metabolism.^6^ Within the nervous system, microbiota-derived metabolites and immune mediators influence gene expression programs associated with neurodevelopment and behavior through the gut–brain axis.^38^ ^,^ ^88^ Future studies employing single-cell and spatial epigenomic approaches will be essential for resolving the cellular specificity of these regulatory interactions.^102^

### 4.4. Tumor microenvironment as a frontier in microbial epigenetics

Recent studies have expanded microbial epigenetics beyond classical host-microbiota systems to include the tumor microenvironment. Intratumoral microbial communities exhibit substantial heterogeneity across cancer types and may contribute to disease progression by modulating host epigenetic and transcriptional programs. Microbial metabolites, inflammatory mediators, and metabolic interactions can influence chromatin remodeling, immune cell differentiation, and tumor cell plasticity.^95^ ^-^ ^97^ Furthermore, emerging evidence indicates that microbial heterogeneity within tumors may contribute to cellular heterogeneity and therapeutic responsiveness through epigenetic mechanisms.^94^ These findings broaden the conceptual framework of microbial epigenetic regulation and highlight the importance of considering complex disease microenvironments when examining host-microbe interactions.^103^

### 4.5. Conclusions and future perspectives

Advances in single-cell sequencing, spatial transcriptomics, metabolomics, and epigenome profiling are transforming the study of host-microbe interactions. Integrating these technologies across diverse taxa will facilitate the identification of conserved and lineage-specific mechanisms through which microbial signals influence host regulatory systems. To further advance the field, future research should not only prioritize mechanistic validation, longitudinal analysis of epigenetic dynamics, and greater representation of non-model organisms, but also pursue targeted studies that manipulate specific microbial taxa or metabolites in controlled experimental models. For instance, the application of CRISPR-based epigenome editing techniques in combination with defined microbial colonization could reveal direct causal links between individual microbial signals and host gene regulatory changes. Furthermore, deploying longitudinal multi-omics analyzes in natural and experimental populations will help disentangle the temporal and causal relationships between microbial community dynamics and host epigenetic states. Such efforts will be essential for understanding how microbial communities shape host phenotypes across ecological, developmental, and pathological contexts.

## Data Availability

No new data were generated for this study.
